# Marital status is an independent prognostic factor in inflammatory breast cancer patients: an analysis of the surveillance, epidemiology, and end results database

**DOI:** 10.1007/s10549-019-05385-8

**Published:** 2019-08-14

**Authors:** Yan-ling Liu, Dun-wei Wang, Zhu-chun Yang, Rui Ma, Zhong Li, Wei Suo, Zhuang Zhao, Zhi-wen Li

**Affiliations:** 1 Department of Oncology, Jilin Cancer Hospital, Changchun, 130012 Jilin China; 2 Department of Anesthesiology, First Hospital of Jilin University, Changchun, 130021 Jilin China; 3 Department of Radiation Oncology, Jilin Cancer Hospital, Changchun, 130012 Jilin China

**Keywords:** Inflammatory breast cancer, Marital status, SEER, Survival

## Abstract

**Objectives:**

The aim of this analysis was to study the impact of marital status on inflammatory breast cancer (IBC) patients, as the prognostic impact is yet to be studied in detail.

**Methods:**

Data of IBC patients from 2004 to 2010 were sorted out from the database of surveillance, epidemiology, and end results (SEER), and overall survival (OS) rates and breast cancer-specific survival (CSS) rates were compared between a group of married and unmarried patients. The comparison was performed by Kaplan–Meier method with log-rank test, and multivariate survival analysis of CSS and OS was performed using the Cox proportional hazard model.

**Results:**

Data of 1342 patients were collected from the SEER database, on an average 52% of married patients (*n *= 698, 52.01%) and 48% of unmarried patients (*n* = 644, 47.99%) for this analysis. Married patients were more likely to be more younger (aged ≤ 56) (52.44% vs. 43.94%), white ethnicity (83.24% vs. 71.58%), HoR positive (48.28% vs. 41.61%), more patients received surgery (78.51% vs. 64.60%), chemotherapy (90.69% vs. 80.12%) and radiotherapy (53.44% vs. 44.41%) compared to unmarried group, and less likely to be AJCC stage IV (26.22% vs. 35.40%) (All *P* ˂ 0.05). Married patients had better 5-year CSS (74.90% vs. 65.55%, *P *< 0.0001) and OS rates (45.43% vs. 33.11%, *P *< 0.0001). The multivariate analysis revealed that marital status is an independent prognostic factor, whereas the data of unmarried patients showed worse CSS (HR 1.188; 95% CI 1.033–1.367; *P *= 0.016) and OS rates (HR 1.245; 95% CI 1.090–1.421; *P *= 0.001).The subgroup analysis further revealed that the OS and CSS rates in the married group were better than the unmarried group, regardless of different AJCC stages.

**Conclusion:**

Marital status was an independent prognostic indicator in IBC patients. As the study reveals, the CSS and OS rates of the married patients were better than those of the unmarried patients.

**Electronic supplementary material:**

The online version of this article (10.1007/s10549-019-05385-8) contains supplementary material, which is available to authorized users.

## Introduction

Inflammatory breast carcinoma (IBC) is a rare clinico-pathological entity of breast cancer. One to six percent of the breast cancer case comes under IBC [1]. According to the tumor-node metastasis (TNM) breast cancer staging system, IBC is classified as T4d and was clinically characterized by diffusing in duration of the skin with an erysipeloid edge, usually with no underlying mass [2]. IBC is also characterized by rapid progression and higher metastatic potential [3]. The 5-year survival rate of IBC patients is much lower compared to other breast cancer patients [4].

In previous researches, the indicators of clinico-pathological characteristics, such as breast cancer subtypes, AJCC TNM stages, tumor sizes, and treatment strategies, were used to predict IBC prognosis and have drawn much attention from the scientists [5–8]. However, social factors are more and more emphasized in the progression of these diseases [9]. It has been revealed that the marital status plays an important role in the prognosis of various carcinomas independently, such as pancreatic carcinoma, prostate carcinoma, lung carcinoma, as well as colorectal carcinoma, where patients in marital status exhibit better survival rates [10–12]. Nevertheless, the comprehension to the effect exerted by marital status on IBC survival rates is still rare.

The surveillance, epidemiology, and end result (SEER) program is supported by the National Cancer Institute (NCI). This program contains research data of 18 different population-based cancer registries that covers 30% of the United States population [13]. The data of SEER have been extensively used for this study, associated between cancerous survival rates and status of marriage among patients [14–16]. In this paper, we have studied the impact of marital status on the IBC survival rates by analyzing the data from SEER database.

## Materials and methods

### Ethical statements

We signed the SEER Research Data Agreement to access the data, using reference number 16462-Nov2016. The data were obtained by means of research methods in accordance with approved protocols. The data analysis was approved by the Office for Human Research Protection to be non-human subjects, who were researched by the United States Department of Health and Human Services, as they were publicly available and de-identified. Thus, it did not require approval by the institutional review board.

### The population of this study

The subjects were selected and determined using the tool of SEER*State v8.3.5 released on March 6, 2018. The time span of the current study was from 2004 to December 2010. The patients chosen for this study were under the following inclusion criteria: (1) primary female IBC patients aged older than 20; (2) IBC diagnosed in line with the International Classification of Disease for Oncology, Third Edition (ICD-O-3; coded as 8530/3). The exclusion criteria were as follows: (1) patients with multiple primary tumors; (2) patients only clinically diagnosed; (3) patients without some important clinico-pathological information, such as AJCC stage, age when diagnosed, race, marital status, and surgical style; (4) patients died within 3 months after surgery; (5) patients without prognostic data. The rest of subjects were enrolled as the initial cohort of SEER.

### Covariables

We analyzed the patients’ characteristics under the following ten parameters: marital status (married, unmarried), age of diagnosis (≤ 56, or > 56 years), race (white, black, or other), Grade (Grade I/II, Grade III/IV, unknown), AJCC stage (IIIA, IIIB, IV), hormone receptor (HoR) (negative, positive, unknown), HER-2 (negative, positive, unknown), surgery (no surgery, partial mastectomy, simple mastectomy, radical mastectomy), chemotherapy(no/unknown, yes), and radiotherapy (no/unknown, yes). The widowed or single (never married or having a domestic partner) or divorced or separated patients were classified as unmarried. The median age of all included patients was 56 years (range: 22–98 years old). Patients were then subdivided into two groups under age criteria: < 56 and ≥ 56 years. Race was again subdivided into white, black, and other (including Asian/Pacific Islander and American Indian/Alaska native). In addition, all chosen cases were restaged in accordance with the 8th AJCC TNM staging classification. The HoR status of the tumor was stratified into HoR estrogen and progesterone-positive (ER +/PR + , ER-/PR + and ER +/PR-) and HoR-negative (ER-/PR-). The definition of ER/PR-positive disease was 1% or greater cells stain positive [17].

The main objective of this study was to compare the CSS and OS rate. The definition of CSS was the duration from tumor diagnosis to the latest follow-up or date of death due to IBC. And the definition of OS was the duration from tumor diagnosis to the latest follow-up or date of death. Of note, the SEER 2016 submission dataset provided a predetermined cutoff date, which contained the data of death till 2014; therefore, the cutoff date was set as December 31, 2014.

### Analyses of statistics

Baseline continuous and categorical variables were presented as median with range and numbers with percentages, respectively. Meanwhile, clinico-pathological characteristics were compared with Fisher’s exact tests or Pearson’s χ^2^. The Kaplan–Meier approach was used to calculate CSS and OS, and the log-rank test was used to compare the variations between different groups. A model of multi-variable COX proportional hazard was established for identification of the independent prognostic factors characterized by the *P* value lower than 0.05 in the log-rank analyses. Statistical significance was set at two-sided *P *< 0.05. The analyses of statistics were conducted using SPSS (SPSS Inc., Chicago, USA, version 23), and the survival curves were generated by GraphPad Prism 5.

## Results

### The characteristics of patients

**Fig. 1:**
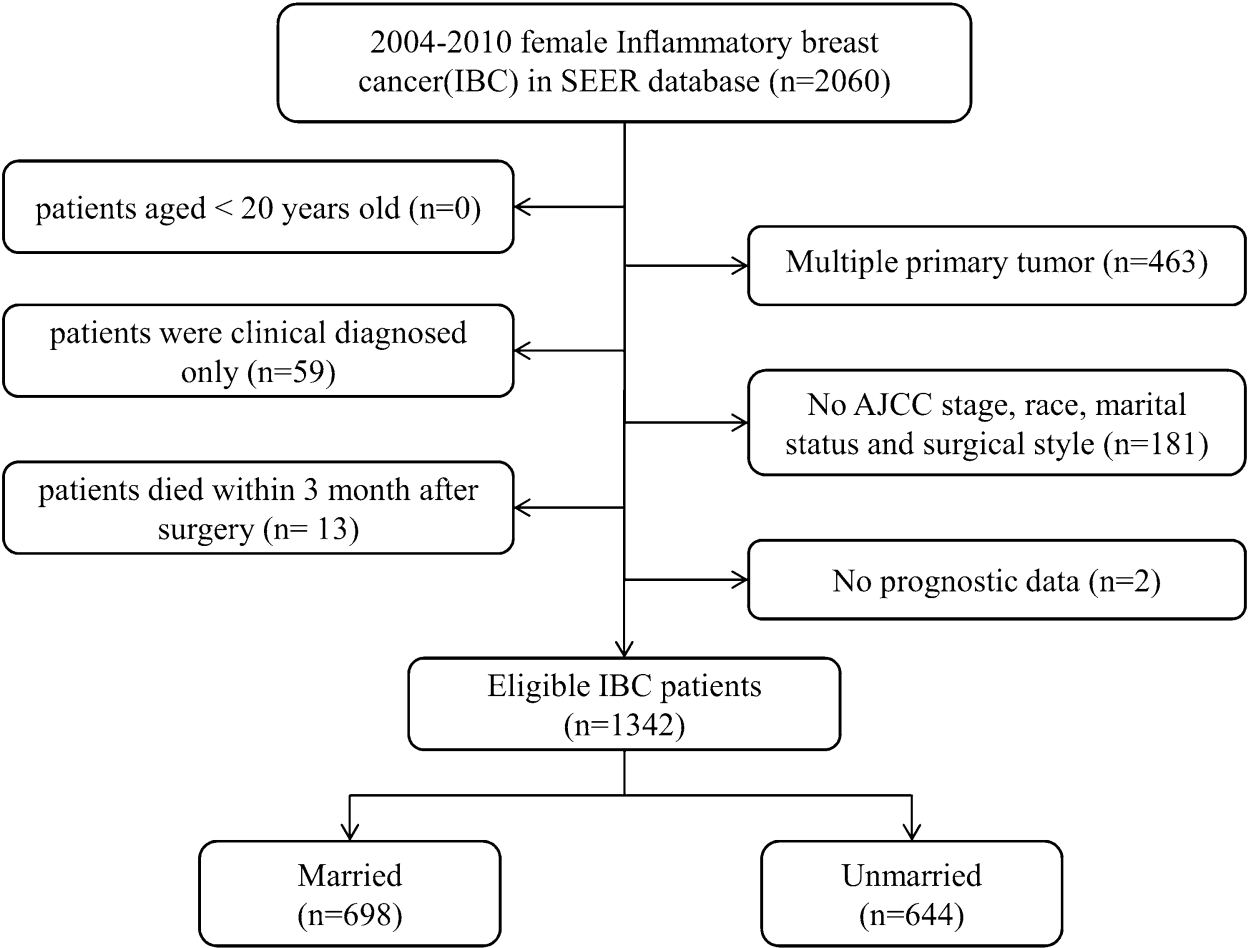
Flow chart for screening eligible patients

We identified 1342 eligible IBC patients diagnosed from 2004 to 2010 in the SEER database with the median follow-up time of 36 months (range: 0–131 months). Patients were further divided into married group (*n *= 698, 52.01%) and unmarried group (*n* = 644, 47.99%), and the detailed process of screening is shown in Fig. 1. The summary of the baseline features of patients in two groups of marriage status is shown in Table 1. There was a significant difference in the age (*P *= 0.002), race (*P *< 0.001), AJCC stage (*P *< 0.001), HoR (*P *= 0.033), rate of undergone surgery (*P *< 0.001), chemotherapy(*P *< 0.001), and radiotherapy (*P *< 0.001) between married and unmarried groups. Married patients were younger (aged ≤ 56) (52.44% vs. 43.94%), white ethnicity (83.24% vs. 71.58%), HoR-positive (48.28% vs. 41.61%), patients undergone surgery (78.51% vs. 64.60%), chemotherapy (90.69% vs. 80.12%), radiotherapy (53.44% vs. 44.41%), and less likely to be in AJCC stage IV (26.22% vs. 35.40%) compared to unmarried group.

**Table 1:** Baseline demographic and tumor characteristics of patients in SEER database

Characteristic	Total	Married	Unmarried	*P* value^+^
	*N* (%)	*N* (%)	*N* (%)	
Age				0.002
≤ 56	649 (48.36%)	366 (52.44%)	283 (43.94%)	
>56	693 (51.64%)	332 (47.56%)	361 (56.06%)	
Race				< 0.001
White	1042 (77.65%)	581 (83.24%)	461 (71.58%)	
Black	228 (16.99%)	78 (11.17%)	150 (23.29%)	
Other*	72 (5.37%)	39 (5.59%)	33 (5.12%)	
Grade				0.160
Grade I/II	262 (19.52%)	141 (20.20%)	121 (18.79%)	
Grade III/IV	773 (57.60%)	412 (59.03%)	361 (56.06%)	
Unknown	307 (22.88%)	145 (20.77%)	162 (25.16%)	
AJCC stage				< 0.001
IIIA	706 (52.61%)	385 (55.16%)	321 (49.84%)	
IIIB	225 (16.77%)	130 (18.62%)	95 (14.75%)	
IV	411 (30.63%)	183 (26.22%)	228 (35.40%)	
HoR				0.033
Positive	605 (45.08%)	337 (48.28%)	268 (41.61%)	
Negative	603 (44.93%)	300 (42.98%)	303 (47.05%)	
Unknown	134 (9.99%)	61 (8.74%)	73 (11.34%)	
HER-2				0.967
Positive	40 (2.98%)	20 (2.87%)	20 (3.11%)	
Negative	69 (5.14%)	36 (5.16%)	33 (5.12%)	
Unknown	1233 (91.88%)	642 (91.98%)	591 (91.77%)	
Surgery				< 0.001
No surgery	378 (28.17%)	150 (21.49%)	228 (35.40%)	
Partial mastectomy	62 (4.62%)	25 (3.58%)	37 (5.75%)	
Simple mastectomy	127 (9.46%)	74 (10.60%)	53 (8.23%)	
Radical mastectomy	775 (57.75%)	449 (64.33%)	326 (50.62%)	
Chemotherapy				< 0.001
No/unknown	193 (14.38%)	65 (9.31%)	128 (19.88%)	
Yes	1149 (85.62%)	633 (90.69%)	516 (80.12%)	
Radiotherapy				< 0.001
No/unknown	683 (50.89%)	325 (46.56%)	358 (55.59%)	
Yes	659 (49.11%)	373 (53.44%)	286 (44.41%)	

*HoR* hormone receptor

*Other includes American Indian/Alaska native, Asian/Pacific Islander, and unknown

^+^The comparison results between married and unmarried group

### Marital status and survival

**Fig. 2:**
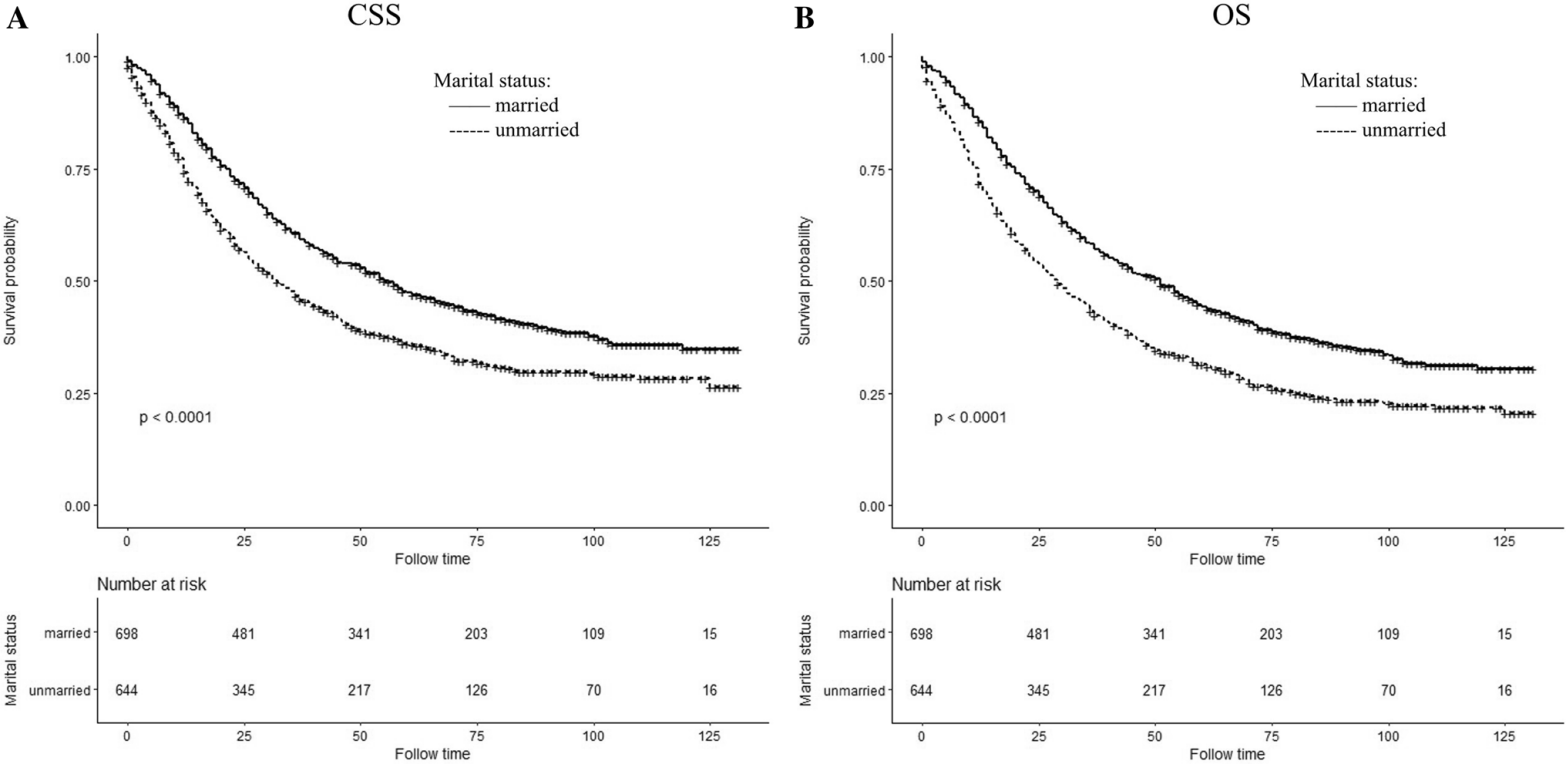
Kaplan–Meier survival curves of cancer-specific survival (**a**) and overall survival (**b**) in different marital status

There were differences in CSS and OS associated with the status of marriage (both log-rank test *P *< 0.0001), and the differences are shown in the Kaplan–Meier curves (Fig. 2). Five-year CSS and OS rate was 47.41% and 44.39% in married patients, 35.98% and 31.52% in unmarried, respectively. Based on the multivariate log-rank test (*P *< 0.05), a few covariates possessed a significant association with CSS that included all the above-mentioned parameters in Section ‘Covariables’. In spite of adjustment by the regression model of COX proportional hazard using these covariables, the results of married status group independently correlated with prognosis and the unmarried group showed significant worse CSS rate than the married group [Hazard ratio (HR)1.188; 95% Confidence interval (CI) 1.033–1.367; *P *= 0.016] (Table 2). The OS rate for marital status also correlated independently as a prognostic factor, and the unmarried group had worse OS (HR 1.245; 95% CI 1.090–1.421; *P *= 0.001] (Table 3).

**Table 2:** Univariate and multivariate survival analysis of CSS in inflammatory breast cancer patients

Characteristic	Univariate analysis		Multivariate analysis		
	Log-rank χ^2^	*P* value	HR	95% CI	*P* value
Marital status	24.239	< 0.001			
Married			Reference		
Unmarried			1.188	1.033–1.367	0.016
Age	2.685	0.101			NI
≤ 56					
>56					
Race	47.365	< 0.001			< 0.001
White			Reference		
Black			1.663	1.398–1.979	< 0.001
Other*			0.781	0.551–1.106	0.164
Grade	14.508	0.001			0.041
Grade I/II			Reference		
Grade III/IV			1.138	0.937–1.381	0.191
Unknown			0.914	0.729–1.147	0.437
AJCC stage	280.068	< 0.001			< 0.001
IIIA			Reference		
IIIB			1.479	1.209–1.810	< 0.001
IV			2.624	2.212–3.113	< 0.001
HOR	68.344	< 0.001			< 0.001
Positive			Reference		
Negative			1.809	1.550–2.111	< 0.001
Unknown			2.185	1.728–2.763	< 0.001
HER-2	7.187	0.028			0.006
Positive			Reference		
Negative			2.208	1.114–4.373	0.023
Unknown			2.588	1.420–4.715	0.002
Surgery	318.410	< 0.001			
No surgery			Reference		
Partial mastectomy			0.493	0.350–0.695	< 0.001
Simple mastectomy			0.495	0.375–0.653	< 0.001
Radical mastectomy			0.484	0.401–0.585	< 0.001
Chemotherapy	64.214	< 0.001			
No/unknown			Reference		
Yes			0.563	0.462–0.687	< 0.001
Radiotherapy	47.991	< 0.001			
No/unknown			Reference		
Yes			0.890	0.765–1.036	0.133

SEER 2004–2010 (*n* = 1342)

*HoR* hormone receptor

*Other includes American Indian/Alaska native, Asian/Pacific Islander, and unknown

**Table 3:** Univariate and multivariate survival analysis of OS in inflammatory breast cancer patients

Characteristic	Univariate analysis		Multivariate analysis		
	Log-rank χ^2^	*P* value	HR	95% CI	*P* value
Marital status	34.333	< 0.001			
Married			Reference		
Unmarried			1.245	1.090–1.421	0.001
Age	12.079	0.001			
≤ 56			Reference		
>56			1.061	0.926–1.215	0.395
Race	50.956	< 0.001			< 0.001
White			Reference		
Black			1.709	1.448–2.017	< 0.001
Other*			0.857	0.624–1.178	0.341
Grade	10.623	0.005			0.055
Grade I/II			Reference		
Grade III/IV			1.089	0.911–1.302	0.348
Unknown			0.888	0.719–1.096	0.267
AJCC stage	267.703	< 0.001			< 0.001
IIIA			Reference		
IIIB			1.448	1.200–1.748	< 0.001
IV			2.399	2.040–2.820	< 0.001
HOR	54.232	< 0.001			< 0.001
Positive			Reference		
Negative			1.656	1.433–1.914	< 0.001
Unknown			1.981	1.585–2.476	< 0.001
HER-2	6.871	0.032			0.008
Positive			Reference		
Negative			1.920	1.019–3.619	0.044
Unknown			2.291	1.319–3.981	0.003
Surgery	29.854	< 0.001			
No surgery			Reference		
Partial mastectomy			0.472	0.339–0.657	< 0.001
Simple mastectomy			0.508	0.391–0.661	< 0.001
Radical mastectomy			0.505	0.422–0.604	< 0.001
Chemotherapy	114.104	< 0.001			
No/unknown			Reference		
Yes			0.499	0.415–0.600	< 0.001
Radiotherapy	59.602	< 0.001			
No/unknown			Reference		
Yes			0.864	0.748–0.996	0.045

SEER 2004–2010 (*n* = 1342)

*HoR* hormone receptor

*Other includes American Indian/Alaska native, Asian/Pacific Islander, and unknown

### Analysis of the effect of marital status according to AJCC stage in subgroups

**Fig. 3:**
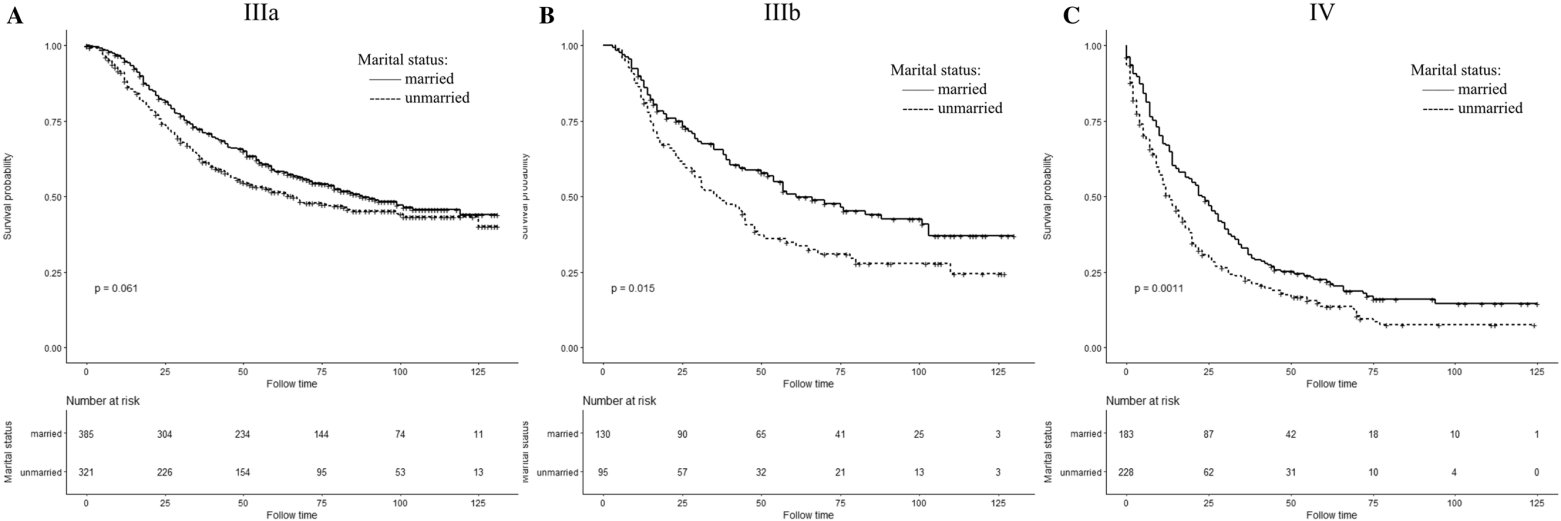
Kaplan–Meier survival curves of cancer-specific survival in different AJCC stage subgroup IBC patients according to marital status

**Fig. 4:**
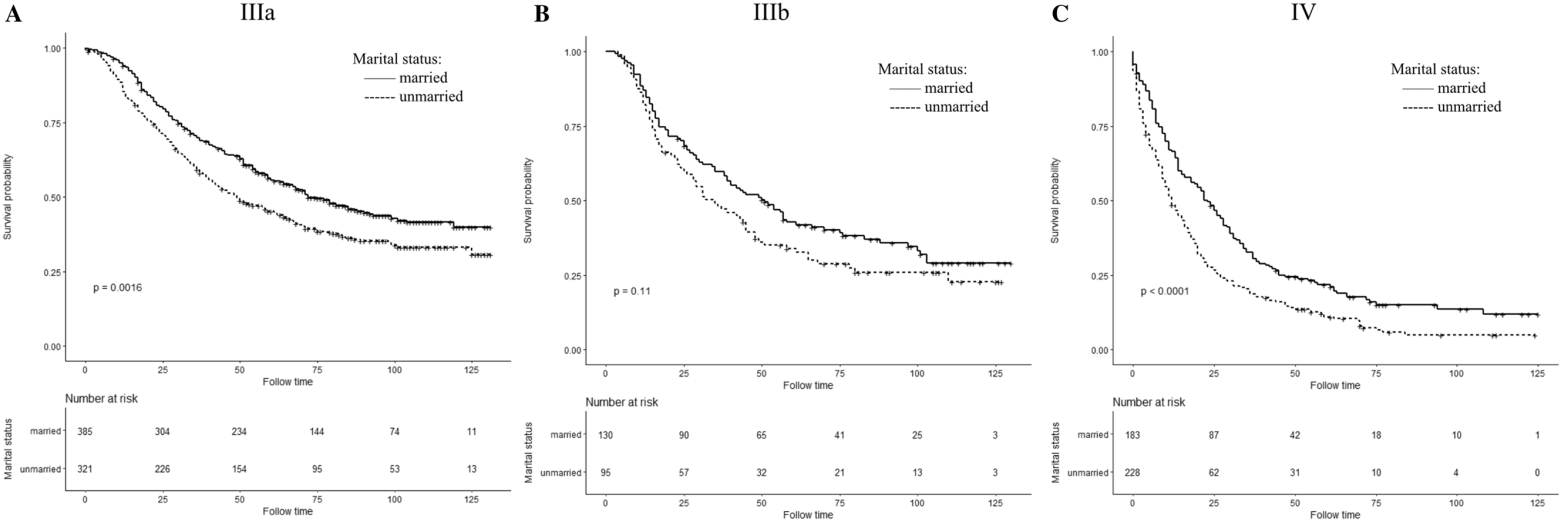
Kaplan–Meier survival curves of overall survival in different AJCC stage subgroup IBC patients according to marital status

We have analyzed the effects of marital status on IBC survival patients at different AJCC stage subgroups. Figures 3 and 4 show Kaplan–Meier curve of CSS and OS rates in different AJCC stages; regardless of the stage, the CSS and OS survival rates of the married group were better than those of the unmarried group. For AJCC stage IIIA patients in married group, the 5-year CSS and OS rates were 58.27% and 55.70%, respectively, in comparison with unmarried group which is 51.60% and 45.21% (in log-rank test, CSS: *P *= 0.061; OS: *P *= 0.0016). In stage IIIB patients, the five-year CSS and OS rates were 50.84% and 42.81% in married patients and 34.92% and 33.83% for unmarried patients(in log-rank test, CSS: *P *= 0.015; OS: *P *= 0.11). The stage IV patients also showed better 5-year CSS and OS rates in married group which is 22.48% and 21.86% compared to the percentage of unmarried patients that is 13.57% and 11.06% (in log-rank test, CSS: *P *= 0.0011; OS: *P *< 0.0001).

## Discussions

This SEER analysis is the first study to specifically examine whether marital status has a significant impact on the survival rate of IBC patients. On analyzing the case history of 1342 IBC patients, we observed a remarkable higher risk of death in unmarried patients than married patients. After the control of demographic tumor characteristics and treatment methods, unmarried patients had 18.8% higher risk of cancer-specific deaths and 24.5% higher risk of overall deaths compared to married patients with IBC.

There are some reports on the association between the prognosis of breast carcinoma and marital status [14, 18–21]. Two studies from M.D. Anderson Hospital did not detect marital status had a significantly effect on survival when stage and the other variables were included. The differences in survival rate in all the covariables always remained higher in married group [18, 19]. To add on to the evidence, many scholars have found that the marital status has a significant impact on the prognosis of other type of breast cancer [20, 21]. Adekolujo et al. found that unmarried females with breast cancer were at greater risk for stage IV disease on diagnosis and more poor outcomes compared to married females [20]. Hinyard L also found that the probability of late-stage diagnosis among unmarried female patients was 1.18-fold higher than that of married female patients. In the analysis after adjustment, unmarried women were more likely to die of breast cancer and more likely to die of all causes than married women, irrespective of all AJCC stages [21]. The results of these studies basically conclude that the prognosis is better and staging is earlier in married patients than that of unmarried patients, which is basically consistent in all the results of our studies. Some scholars even found that the survival benefit associated with marriage was larger than the published survival benefit of chemotherapy [14].

The influence of marital status on prognosis may be related to tumor stage, proportion of patients receiving treatment, and social support [14, 20–22]. Our study found that married patients who were in earlier tumor stage were younger and had significantly higher proportion of white race and HoR-positive. In addition, we found that the likelihood of receiving surgeries, chemotherapy, and radiotherapy among unmarried subjects was lower than married ones. Hershman et al. also stated that unmarried subjects tend to postpone the onset of adjuvant chemotherapeutic treatment after receiving surgeries of breast carcinoma, which resulted in increased mortality rate [23]. This partly explains why unmarried patients have poorer prognosis.

However, after the adjustment of these factors, multivariate analysis still found that marital status was an independent prognostic risk factor for patients with IBC. Thus, there are deeper reasons to how the marital status affects the prognosis of IBC patients. Patients who are unmarried lack the support and care from spouses, thus often suffer from distressed psychological state and indulge in bad habits, (smoking and excessive drinking) which cause development of tumor and under-treatment of diseases [24–26]. The patients in marital status are supported economically and encouraged by their spouses, which conduce to the acceptance for adjuvant therapies and surgeries, thus leading to the differentiation to a certain degree [27, 28].

Our study is interpreted with caution due to the limited access of the SEER database. First, the information of marriage status obtained from the database was collected when patients were diagnosed of IBC, which could have been possibly changed during the time span of follow-ups, thus probably affecting the final results. Second, no accurate details of marriage were provided by the database of SEER, which was capable of influencing the results of survival rates [29]. Third, the therapeutic details are limited in the database of SEER, especially radiotherapy and chemotherapy. In spite of the above-mentioned constraints, it is indicated by the current study that the status of marriage has a significant impact on the survival of IBC. The significance of this work is in studying the essential and continuous effects exhibited by the marital characteristics, especially social supports, after detecting and treating carcinoma and the survival rates. The probability of curing unmarried patients has been remarkably increased by investment in social support intervention. Further investigation in analysis such as cost and benefits and the intervention approaches that are innovative and cost-effective could be an effective way of improving carcinoma prognosis of unmarried patients.

## Conclusion

To conclude, our study has revealed that marital status is an independent prognostic indicator of IBC patients and it has a significant impact to extend the CSS and OS rate. According to our analysis on different variables between married and unmarried patients, married patients have better CSS and OS than unmarried patients. Going forward, further analysis on the data of married patients can throw more light on the reasons for their extended CSS and OS, which could help the unmarried patients to fight against the IBC and increase their CSS and OS.

## Electronic supplementary material

Below is the link to the electronic supplementary material.

Supplementary material 1 (XLSX 788 kb)

## Data Availability

The data that support the findings of this study are available from SEER but restrictions apply to the availability of these data, which were used under a signed SEER Research Data agreement for the current study, and so are not publicly available. Data are however available from the authors upon reasonable request and with permission of SEER
